# Remarks on the External Use of Cold Water in Fever

**Published:** 1801-01

**Authors:** T. Garnett

**Affiliations:** Professor of Natural Philosophy and Chemistry in the Royal Institution of Great Britain.


### *Remarks on the external Uſe of cold Water in Fever*; *by* T. Garnett, M. D. Profeſſor of Natural Philoſophy and Chemiſtry in the Royal Inſtitution of Great Britain.

NO greater improvement has lately taken place in medicine than in the treatment of Fevers, by the external uſe of cold water; a mode which was firſt, I believe, practiſed by Dr. Wright, but ſince elucidated by the acute reaſoning and confirmed by the experience of Dr. Currie. I have ſeveral times witneſſed, not without ſome degree of aſtoniſhment, the wonderful efficacy of this plan; and I only preſume to ſtate a few facts, with a view of drawing the attention of the faculty in this this city to this point; for, though the plan is ufed by fome very judicious practitioners, it is by no means general. I am, however, convinced from experience, that there are few cafes of *typhus* in which the pulfe exceeds 100, where the fkin is dry, and its heat confiderably above the natural heat of the human blood, (circumftances which ought to be diligently attended to) where a cure may not fpeedily be effected by it, provided it be employed within the firft fix or eight days from the attack of the fever. Among many other cafes, I fhall only give the following:

I was defired to vifit Mr. T. a refpectable manufacturer in Glafgow, who laboured under a mild kind of typhus, from which, however, he did not recover fo quickly as was expected. It was on the twentieth day of the fever that I faw him, and I prefcribed for him the oxymuriat of potafh, a remedy which I have been in the habit of ufing in this complaint for fome years with great fuccefs. He grew better daily; but before he was perfectly recovered, Mrs. T. who had given him almoft conftant attention, was attacked with fymptoms of fever; I did not fee her till the third day after the attack, when I found her pulfe not lefs than 130; the heat of her fkin 106°, without any moifture; her eyes had a confiderable degree of wildnefs, and her tongue was quite brown and parched. In fhort, I think I had never feen the difeafe at the fame period attended with worfe fymptoms.

As this feemed a cafe exactly adapted to the plan laid down by Dr. Currie, I propofed it to the furgeon who attended her along with me, a very intelligent practitioner, who immediately affented to it. She was directed to be taken out of bed, and placed in a large tub on a ftool, and a large bucket of cold water, in which about a pound of common falt had been diffolved, was thrown upon her; her fkin was immediately wiped dry, and fhe was put to bed. In about ten minutes afterwards I went into the room, and found the pulfe 94; the heat of the fkin 96°. On afking her how fhe felt herfelf, fhe replied, as well as ever fhe was in her life: I left her, directing the affufion to be had recourfe to again if the febrile fymptoms returned during the night. I faw her the next morning, and found her without any fever, of which fhe had no return.

Another patient on whom I tried this remedy, was Mifs R. aged 17. She had taken the infection from her brother, who had recovered very flowly from a typhus of the worft kind. She was attacked with fhivering, fucceeded by great heat, and pain in the back; her pulfe was 128; the heat of the fkin 102°, and dry; her tongue parched and brown, though not quite fo bad as in the former cafe. The cold water was ufed in the ſame manner, and in a quarter of an hour the febrile ſymptoms had vaniſhed; the pulſe was under 100, and the heat of the ſkin natural. The febrile ſymptoms, however, returned in about ſix hours, but went off on repeating the affuſion; they returned again in the courſe of the next day, but were again overcome; ſhe was obliged to uſe the affuſion five times; but at laſt, after forty-eight hours from the firſt affuſion, the complaint had totally left her.

Two days after this, another brother of Miſs R's was ſeized with ſymptoms of fever, in which the heat of the ſkin, pulſe, and tongue, were much the ſame as in his ſiſter's caſe; and he was delirious, conſtantly wiſhing to go to the grammar ſchool to receive a prize to which he was entitled. The affuſion was uſed in the ſame manner as in the preceding caſes; the febrile ſymptoms immediately vaniſhed—the delirium left him—his pulſe and heat became natural—he fell into a ſound ſleep—perſpired profuſely; and in the morning found himſelf ſo well, that I am pretty ſure he did go to the grammar ſchool to claim his prize.

*Royal Inſtitution, Dec.* 1, 1800.

